# The SmartNTx-study: a prospective, randomized controlled trial to investigate additional interventional telemedical management versus standard aftercare in kidney transplant recipients

**DOI:** 10.3389/fneph.2025.1591962

**Published:** 2025-06-19

**Authors:** Mario Schiffer, Lars Pape, Julia K. Wolff, Raoul Gertges, Vanessa Visconti, Karen Reichert, Anja Pfau, Anne Dieterle, Katja Sauerstein, Andreas Kribben, Kristina Boss, Sinem Karaterzi, Felix Nensa, Philipp Winneckens, Mario Cypko, Wiebke Duettmann, Bianca Zukunft, Eva Schrezenmeier, Marcel G. Naik, Fabian Halleck, Roland Roller, Sebastian Möller, Oliver Amft, Klemens Budde

**Affiliations:** ^1^ Department of Nephrology and Hypertension, University Hospital Erlangen, Erlangen, Germany; ^2^ Department of Pediatrics II, University Hospital of Essen, University of Duisburg-Essen, Essen, Germany; ^3^ Institut für Gesundheits- und Sozialforschung (IGES) Institute, Berlin, Germany; ^4^ Department of Prevention Research and Social Medicine, Institute for Community Medicine, University Medicine Greifswald, Greifswald, Germany; ^5^ Department of Nephrology, University Hospital of Essen, University of Duisburg-Essen, Essen, Germany; ^6^ Institute for Artificial Intelligence in Medicine (IKIM), University Hospital Essen, Essen, Germany; ^7^ Intelligent Embedded Systems, University of Freiburg, Freiburg, Germany; ^8^ Hahn-Schickard, Freiburg, Germany; ^9^ Department of Nephrology, Charité, Berlin, Germany; ^10^ German Research Center for Artificial Intelligence (DFKI), Berlin, Germany; ^11^ Technische Universität Berlin, Berlin, Germany

**Keywords:** kidney transplantation, aftercare, patient empowerment, APP, randomized controlled trial (RCT)

## Abstract

**Background:**

Regular follow-up care after kidney transplantation is performed in transplant centers together with local nephrologist practices in Germany. Patients after kidney transplantation have to fulfill many tasks and manage their disease, follow a complex therapeutic regimen, communicate with the transplant center and home nephrologists, and coordinate doctor appointments. It has been shown that mHealth solutions such as mobile phone applications (apps) can support patients in their self-management. However, stand-alone apps have limitations and ideally, the mHealth solutions are embedded in a holistic treatment approach, including healthcare professionals.

**Methods:**

We will conduct a 1-year, prospective, randomized, 2-armed, parallel group multicenter trial in three German Kidney Transplant Centers (KTCs) to demonstrate that additional and continuous interventional telemedical management will improve health after kidney transplantation in patients of all ages. Therefore, a composite endpoint of seven key outcome variables [fewer hospitalizations, shorter length of hospitalization, less development of *de novo* donor-specific antibody (DSA), better medication adherence, lower tacrolimus intra-patient variability, better blood pressure control, and better renal function after kidney transplantation]was defined. All the patients will receive the same routine post-transplant aftercare. The patients in the interventional arm will receive additional predefined telemedical management, including regular telemedicine visits and automatic bidirectional data transfer (e.g., vital signs, wellbeing, medication plan, and laboratory data together with a chat option) between the patient at home and the KTC through a certified smartphone app. If necessary, a home nephrologist can be included in the automatic data transfer. In the interventional arm, the iBox score will be used to better detect patients at risk for early graft failure and drug-drug interactions will be regularly checked with certified software.

**Discussion:**

The study aims to prolong patient and graft survival through additional telemedical services in order to reduce avoidable hospitalizations, improve treatment of co-morbidities, and improve adherence through patient empowerment, which should result in lower health care costs, and better quality of life of patients after kidney transplantation.

**Clinical Trial Registration:**

ClinicalTrials.gov, identifier NCT05897047.

## Introduction

End-stage renal disease (ESRD) and kidney replacement therapies are linked to high morbidity and mortality (1). Compared to dialysis, kidney transplantation (KTx) offers the best survival, quality of life (QoL), and lower healthcare costs for patients with ESRD. According to current guidelines for immunosuppressive therapy, tacrolimus (a calcineurin inhibitor) combined with anti-IL-2 receptor antibodies (e.g., basiliximab), mycophenolate (e.g., Mycophenolate mofetil (MMF) or Enteric coated Mycophenolate sodium (EC-MPS)), and steroids, has significantly improved renal transplantation outcomes, particularly in reducing acute rejection rates to below 15% (2–5). As rejection is most common in the first year, early prevention is crucial for optimal outcomes, as rejections are linked to graft loss, prolonged hospitalizations, and over-immunosuppression. In addition, over-immunosuppression can increase infection risks, such as urinary tract infections (UTI), BK virus (BKV), and cytomegalovirus (CMV) (6–13). Thus, an adequate initial rejection prophylaxis is the key to optimal long-term outcomes in KTx.

Immunosuppressive drugs can also elevate cardiovascular risks, such as hypertension and diabetes, necessitating personalized treatment (14–19). The leading cause of graft loss remains death with a functioning graft, followed by T-cell mediated rejection (TCMR) and chronic antibody-mediated rejection (ABMR), often linked to non-adherence (20). Holistic care addressing adherence and cardiovascular risks through structured aftercare, adherence training, and sports therapy has been shown to improve graft survival (8, 21). This holistic approach, which systematically addresses non-adherence and cardiovascular risks in a structured aftercare program with adherence training and sports therapy, can lead to an improvement in graft survival, as demonstrated in our recent publication (22).

Yet, cardiovascular death remains the most common cause of premature death, with hypertension as the major modifiable risk factor (23–25). Despite improvements, blood pressure control and cardiovascular risk factor management after KTx remain suboptimal (26). In the multicenter “FAVORIT” trial, 44% of patients on antihypertensive medication had blood pressure readings above 140/90 mmHg (27). Furthermore, 24-hour blood pressure monitoring has shown better correlations with end-organ damage than office measurements, and the 2018/2023 ESC/ESH Guidelines recommend a target of <140/90 mmHg for patients over 65 (18, 28). The treatment goal here should not differ from that of the dialysis population or patients with advanced CKD. A target of less than 135/90 mmHg seems a reasonable compromise to avoid graft hypoperfusion and cardiovascular complications (18). For children, blood pressure targets should match age-related norms, with hypertension defined as values above the 95th percentile + 5 mmHg (29). German normal blood pressure percentiles for children and adolescents are given in the KIGGS-Report (30).

Since renal function alone has limitations in predicting graft loss, *Loupy* et al. developed an AI-based model called iBox to predict graft loss better in kidney transplant recipients (KTRs). The model integrates data such as transplant time, renal function, proteinuria, donor-specific antibodies (DSAs), and histopathology, achieving excellent predictive accuracy (C index 0.81). It has been validated across European and US cohorts and is proposed as an early surrogate endpoint for clinical trials (31–33). The iBox is an integrative, accurate, and readily implementable risk prediction evaluation for kidney allograft failure, which shows generalizability across centers worldwide and common clinical scenarios. The iBox risk prediction model is used as a valid and early surrogate endpoint in clinical trials.

We and others have shown that non-adherence is another common problem after kidney transplantation and thus has been called the 5th vital sign (34–36). There are many tools to evaluate medical adherence, including the level to which a disease (e.g., blood pressure) is controlled, assessment of drug levels, and self-assessment by the patient or the physician in charge, amongst others. In general, structured self-assessments are considered to be very useful and provide an important cornerstone for assessing adherence. The Basel Assessment of Adherence to Immunosuppressive Medication Scale (BAASIS^©^) has been developed in particular to detect immunosuppressive medication adherence and is increasingly used (37–41) Evidence suggests that tacrolimus intra-patient variability (IPV) is a strong prognostic factor for long-term outcomes, with high IPV linked to poor results. Reducing IPV requires addressing non-adherence through patient education and minimizing drug-drug interactions (42, 43). In general, drug-drug interactions are a frequent problem in patients on complex medication schemes. Unsurprisingly, they may cause severe side effects and hospitalizations (42, 44–46). In tacrolimus-treated organ transplant recipients, the inadvertent application of enzyme-inducing drugs may cause acute transplant rejections, while inhibition of drug metabolism may cause severe toxicity in nephrotoxicity (47). Thus, regular and easy assessment of drug-drug interactions, e.g., by a certified software solution, may be useful and may help to reduce tacrolimus IPV and drug-associated complications.

UTIs are common after transplantation, with approximately 74% of patients affected in the first year post-transplant, decreasing to 35% in the second year and 21% by the fourth year (48, 49). Severe UTIs are a frequent cause of hospitalization and Abbott et al. found that late UTIs increased the risk of graft failure 2.35-fold (50). Moreover, recipients who develop septicemia are at higher risk of death due to cardiovascular events compared to recipients without infections. Pellè et al. confirmed that late UTIs were associated with worse long-term patient survival and are an independent risk factor for worse outcomes (49). Late UTIs are also associated with higher mortality from cardiovascular causes, and recurrent UTIs in the first year post-transplant predict worse graft function at 3 years (HR 2.2; 95% CI 1.3–3.5) (51).

In conclusion, long-term success after transplantation depends on multiple factors. A holistic approach with regular follow-up from experienced physicians is vital. Patients face challenges managing complex regimens, coordinating with healthcare providers, and maintaining adherence. mHealth applications offer a promising solution to support self-management and empower patients, with studies demonstrating the utility of eHealth and telemedicine (52–54).

In this randomized trial, we test whether a comprehensive eHealth intervention in combination with a dedicated team will have a positive impact on adherence, blood pressure control, hospitalizations, and transplant outcomes.

## Methods and analysis

**Figure 1:**
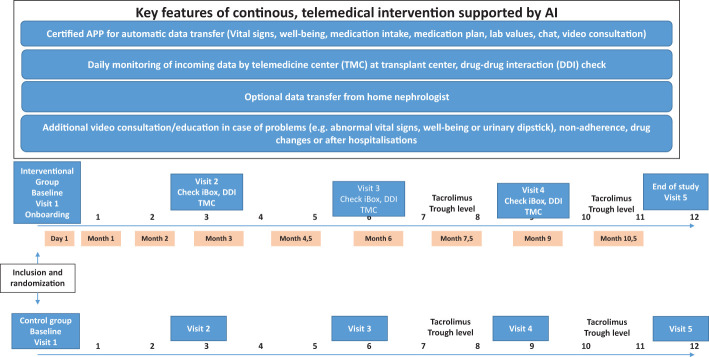
Study design.

In this 1-year, prospective, randomized, 2-armed, parallel group multicenter trial in three German Kidney Transplant Centers (KTCs), we want to demonstrate that additional and continuous interventional telemedical management will lead to a better composite endpoint of seven key outcome variables. The key outcome variables are fewer hospitalizations, shorter length of hospitalization, less development of *de novo* DSA, better medication adherence, lower tacrolimus intra-patient variability, better blood pressure control, and better renal function after kidney transplantation. **Figure 1** depicts the study design.

All patients will receive the same routine post-transplant aftercare. Patients in the interventional arm will receive predefined additional telemedical management, including regular telemedicine visits and automatic data transfer (vital signs, wellbeing, medication plan, laboratory data, and chat) between the patient at home and a KTC through a certified smartphone application. Furthermore, the home nephrologists of patients in the interventional arm will be invited to participate in the automatic data transfer of key variables (such as vital signs and laboratory data) with the KTC. A separate telemedicine team will constantly review the incoming data according to a predefined protocol and eventually contact the patient and/or the home nephrologist to start appropriate interventions. For patients in the interventional arm, physicians will use the certified iBox score based on the “iBox” algorithm (Predict4Health, Paris, France) to better detect patients at risk for early graft failure. In the interventional arm, drug-drug interactions will be regularly checked with the help of certified software (ID Medics^®^, GmbH & Co. KGaA, Berlin, Germany).

It has been previously demonstrated that additional telemedical care may affect a variety of important key outcome variables for long-term success after kidney transplantation (22, 52, 53). In our trial, we chose a composite endpoint of such variables as the primary outcome. Under the assumption of a small to medium effect size of Cohen’s d = 0,31, we calculated that 192 patients per group (including a 20% drop-out rate) are needed to show a difference between groups. Thus, the study population will consist of approximately 384 KTRs, irrespective of age. They will undergo central web-based randomized placement in one of the two treatment groups in a 1:1 ratio. Because expected key outcomes (e.g., hospitalization rate and adherence) may differ according to the age of participants and the time post-transplant, we will stratify them at randomization not only by KTC, but also according to patients’ age: 18–65 years and >65 years. Randomization of children was stratified only by age and not by KTC because of the low expected number of children per KTC (< 12 years, 12 to 18 years).

### Method of assigning patients to the treatment groups

At the baseline visit, study personnel will register the patients, who will sign the ICF, in the database and will assign a unique patient identification number (PID number). The PID number will only identify the patient’s records. The investigator will keep a master patient list on which the PID number and the full name, address, and telephone number of each patient are listed.

For data collection, five study visits at the KTC are scheduled (months 0, 3, 6, 9, and 12). For the determination of tacrolimus trough level, two additional time points will be used (months 7.5 and 9.5). In addition, medication adherence, quality of life, and treatment satisfaction will be assessed by validated questionnaires.

### Group 1 – routine aftercare

The routine aftercare (control group) will be scheduled according to the current standard of care, which depends on the time after transplantation, medical condition, and other individual factors. Standard immunosuppression will be applied according to international recommendations (55). The prophylaxis and treatment of infections will follow the current standard of care as outlined in recent guidelines (56–61), e.g., *Pneumocystis jirovecii* pneumonia (PjP) prophylaxis, CMV prophylaxis according to guidelines, or regular post-transplant BKV monitoring according to guidelines (3). Screenings for antibodies against human leukocyte antigen (HLA) will be performed once within the first 3 months (HLA screening results prior to randomization of the patient can be used if these are not older than 6 months) and after 1 year and in the case of suspected rejection (62). Overall medical treatment of the KTRs will be performed according to KDIGO guidelines (3). A kidney biopsy should be performed in patients with suspected rejection and classified according to the most recent Banff criteria (63). An integral part of the current routine aftercare is regular home nephrologist and KTC visits in adults, where data are captured in an electronic health record (EHR) (64). **Table 1** provides the proposed schedule for post-transplant aftercare. The proposed schedule will be adapted to the patient´s individual needs.

**Table 1:** Routine visits after transplantation.

Time of visit	Time point
Month 1	1x per week after discharge (either at KTC or by home nephrologist)
Month 2	1x per week (either at KTC or by home nephrologist)
Month 3	1x per 2 weeks (either at KTC or by home nephrologist)
Months 4 to 6	1x per 3 weeks (either at KTC or by home nephrologist)
Months 6 to 12	1x per month (either at KTC or by home nephrologist)
After month 12	1x per 4–6 weeks (either at KTC or by home nephrologist)

### Group 2 - intervention group

KTRs allocated to the intervention arm will receive identical routine post-transplant aftercare as the patients in the control group (see above, **Table 1**). In addition, they will receive predefined interventional telemedical management by a separate telemedicine team as outlined above. Participants in the telemedicine group will have a certified app (comjoodoc EASY run by Carealytix Digital Health GmbH, Valley, Germany) that allows automatic data transfer (e.g., vital signs, wellbeing, medication plan, laboratory data, and chat) via a smartphone or tablet between the patient and a KTC (54). To increase the comfort of self-monitoring and easy documentation, the patients will be offered devices that directly transfer data into the smartphone app via standard Bluetooth connectivity. Moreover, participants in the intervention group will receive urine dipsticks with appropriate education to support early diagnosis of urinary tract infections at home. Finally, the patients will receive extensive education on medical therapy, counseling on adherence, and information on telemedicine management. Home nephrologists willing to participate and equipped with appropriate software (e.g., Nephro7 by MedVision AG, Unna, Germany) may participate in automatic data exchange of key variables (such as vital signs and laboratory data) with the KTC.

### Inclusion criteria

- Kidney transplantation within the last 12 months
- Treatment with tacrolimus
- Routine aftercare planned at a KTC
- Ability to use a smartphone or tablet, or with the help of someone close by
- For children < 12 years, parents have to take over the use of the smartphone
- Patients who are willing and able to participate in the study and from whom written informed consent has been obtained prior to study participation or pediatric patients with parental consent
- Ability to communicate in German or English
- Adequate and stable renal function (eGFR > 30 ml/min, proteinuria < 1g/g creatinine if not pre-existing before KTx); eGFR will be determined according to CKD-EPI for adults or Schwartz formula for children

### Exclusion criteria

- Patients with mental dysfunction or inability to comply with the study protocol or pediatric patients whose parents cannot comply with the study protocol
- Any significant diseases or clinically significant findings, including psychiatric and behavioral problems, medical history, and/or physical examination findings that would, in the opinion of the investigator, preclude the patient from participating in the study
- History of alcohol or drug abuse with less than 6 months of sobriety
- Participation in any other interventional clinical trial less than 1 month before participation in this study
- Patients who have been institutionalized by official or court order
- Patients with a combined kidney transplant or multi-organ recipients [other solid organ (e.g., pancreas) or bone marrow]
- Presence of DSA with MFI > 1,000 at time of transplantation
- Recurrence of underlying kidney disease [e.g., focal segmental glomerulosclerosis (FSGS) or atypical hemolytic uremic syndrome (aHUS)]
- Patients with active malignancy post-transplant with the exception of local, non-invasive, fully excised, cutaneous basal cell carcinoma, cutaneous squamous cell carcinoma, or cervical carcinoma *in situ*
- Patients with clinically symptomatic congestive heart failure (CHF) or symptomatic coronary artery disease
- Patients with documented [either by serology and/or nucleic acid testing (NAT)] clinically active infections [e.g., with a known hepatitis B (HBV), hepatitis C (HCV), HIV, CMV, or BK virus infection]

### Telemedicine team

A separate telemedicine team will constantly review the incoming data according to a predefined protocol, as outlined in **Tables 2**, **3**, and eventually contact the patient and/or the home nephrologist to start appropriate interventions. The team consists of experienced nurses/case managers together with physicians in the KTC. For patients in the intervention arm, physicians will use the certified iBox algorithm (Predict4Health, Paris, France) to better detect patients at risk for early graft failure. Physicians and the telemedicine teams will also use certified software (ID Medics ^®^, ID GmbH & Co. KGaA, Berlin, Germany) to check drug-drug interactions easily.

**Table 2:** Treatment ranges of vital signs and wellbeing in adults.

Characteristic	Critical	Suspicious	Normal	Suspicious	Critical
Systolic blood pressure [mmHg]	<90	<100	100 – 129	130 – 180	>180
Diastolic blood pressure [mmHg]	<50	50 – 59	60 – 89	90 – 100	>100
Heart rate [bpm]	<50	50 – 59	60 – 89	90 – 120	>120
Temperature [°C]	<33.5	33,5 –3 6,2	36,3 – 37,4	37.5 – 38,0	>38.0
Weight change, 1 day [kg]	>(–1,5)	(-1,5) – (-0,5)	+/-0.5	0.5 – 1.5	>1,5
3 days [kg]	>(-2,5)	(-2,5) – (-1,0)	+/-1.0kg	1.0 – 2.5	>2.5
8 days [kg]	>(-3.0)	(-3,0) – (-1,5)		1.5 – 3.0	>3.0
Wellbeing [points]			1 – 2	3 – 4	5

**Table 3:** Telemedicine aftercare tasks.

Prioritization	Nurse	Physician	Senior nephrologists	Local nephrologists
1.	Reports critical vital signs to the physician	Contacts patients with critical values	Guides critical cases	Receives data from the transplant center
2.	Informs the physician on duty	Discusses critical cases with the transplant team’s senior nephrologist	Support for clinical questions	Reviews incoming data
3.	Reports critical values to the physician	Takes action if needed (e.g., contacts local nephrologist or emergency room)	Reviews problematic cases	Performs the onboarding process for new patients
4.	Reviews wellbeing	Reviews problematic cases with the telemedicine nurse	Contact local nephrologists	Can call the telemedicine team in case of technical problems
5.	Calls patients if they are not feeling good	Reviews cases with the transplant team and the senior nephrologist	Reviews critical drug-drug interactions	Can call telemedicine team in the case of medical questions
6.	Discusses critical patients with the physician on duty	Reviews incoming messages and laboratory data	Reviews critical iBox results	May receive calls from telemedicine team regarding problematic patients
7.	Reviews less critical vital signs	Follows problematic cases	Participates in case conferences	Can discuss problematic patients with the telemedicine team, transplant center, or senior nephrologist
8.	Reviews patients with missing data	Answers incoming calls from patients and local nephrologists		May receive re- training
9.	Calls patients who did not transfer data according to the schedule	Trains and re-trains local nephrologists and patients		May participate in case conferences
10.	Discusses problematic cases with the physician on duty	Reviews drug-drug interactions reviews iBox scores		
11.	Reviews normal vital signs			
12.	Answers incoming calls from patients and local nephrologists			
13.	Reviews incoming medical messages	Onboarding of patients		
14.		Organizes and participates in case conferences		
15.	Onboarding of patients			
	Participates in case conferences			

### Onboarding procedure and training of participants

After randomization, a detailed training and onboarding process with the patient will be performed [65], including the following: the app solution is installed on a smartphone and the patient receives instructions from the telemedicine team on how to use the app and how to document vital signs and medication changes. Patients are offered certified devices (scale, blood pressure monitor, and thermometer), which optionally allow them to directly transfer data to a tested and secure mobile software application via Bluetooth connection to increase the ease of self-monitoring. Participants receive urine dipsticks along with appropriate instructions for when to use and how to react in case of symptoms of urinary tract infection. The medication plan is reviewed in the app to ensure correct transmission. The patient will receive instructions on how to document drug intake. With the help of the telemedicine team, the use of the mobile chat messenger and the video system is tested and participants will be instructed to contact the telemedicine team in case of medical or technical questions. Patients will be instructed on the availability of the telemedicine team during regular working hours only and how to handle emergency situations.

### Daily monitoring of incoming data

The patient will document their vital signs, wellbeing, and drug intake in the mobile software application. Data will then be transferred to the telemedicine team in the KTC (54), who will review all incoming data from 8 am to 4 pm on weekdays in a structured way as described in **Table 2**, according to predefined thresholds (54) as outlined in **Table 4**. If the values are outside the critical range, the telemedicine team will contact the patient to clarify the situation and take appropriate action. As outlined in **Table 2**, the telemedicine team may also discuss the medical issue with the KTC or home nephrologist. In the case of a medical emergency or critical data, the telemedicine team will organize appropriate and fast medical help in an emergency unit, eventually with the help of the KTC or home nephrologist.

**Table 4:** Description of the telemedicine service.

Telemedicine services, app	Telemedicine team
Any time	8 am – 4 pm on working days
Transmission and documentation of vital signs, wellbeing, blood sugar (for diabetic patients), and daily steps	Review of vital signs, laboratory values, and wellbeing on working days
Display of medication plan	Review of medication changes
Display of laboratory values	Medical hotline
Tracking of medication intake	Review of adherence
Reminder of medication intake	Recognition of non-adherence
Messages to transplant center	Intervention and individualized lessons
Video consultations with transplant center	Phone calls and medical messages (questions, problems, assistance, receipts, appointments, etc.)
	Semi-structured onboarding of patients (incl. technical aspects, education, self-assessment, important symptoms, medication plan, and handling of medical emergencies)
	Review of drug-drug interactions, iBox score
	Technical support for patients and home nephrologists

Acute medical problems and symptoms and emergency care remain unchanged and are provided by physicians on call, home nephrologists, and emergency rooms.

The telemedicine team will also review the patient’s regular drug intake and whether the patient follows the individual therapeutic plan, which was agreed upon during the onboarding procedure. If the patient’s adherence (either to drug intake or therapeutic plan) decreases, the telemedicine team will get in touch with the patient and assess reasons for non-adherence. Eventually, the therapeutic plan will be adjusted to the needs of the patient. Some patients may be educated again on the importance of adherence and how to implement it in their daily lives. If necessary, the telemedicine teams may repeat this educational session and organize a face-to-face meeting or discuss other supportive measures.

### Sharing of laboratory values

Patients in the intervention group will receive the lab results of key data, such as renal function or tacrolimus trough levels, directly in the app to increase awareness and empower the patients. In addition, key laboratory data may be exchanged with the home nephrologists, who are willing to participate and use an appropriate patient documentation system (e.g., Nephro7, MedVision AG, Unna, Germany). This means that more longitudinal data will be available for the treating physician at the KTC and home nephrologist, which may allow more timely detection of critical situations and better coordinated care.

### Case conferences

The telemedicine team will perform regular case conferences on a monthly basis. During these conferences, difficult cases and important results from drug-drug interaction checks or abnormal findings in iBox will be discussed together with the physicians of the KTC and home nephrologists.

### Certified decision support for individual patient risk assessment and allograft survival prediction

The certified iBox^®^ software will be used for allograft survival prediction and assessment of the individual patient risk for graft loss at each study visit. In the case of abnormal results, an internal review with senior physicians will take place to discuss potential interventions and/or close follow-up monitoring.

### Regular assessment of drug-drug interactions

The telemedicine team will use the certified software ID Medics^®^ for participants in the intervention group to assess potentially harmful drug-drug interactions regularly. The current medication plan will be transmitted directly to the patient app. Any medication changes by the KTC will release a new plan, which will again be transferred to the patient directly. Important findings will be discussed with the senior nephrologist in the KTC and during case conferences.

### Randomization

Randomization will be performed using a predefined variable block randomization scheme, stratified by the participating centers and the age of the participant. Due to low expected patient numbers in the age groups of < 12 years and 12 to 18 years, patients in these age groups will be randomly stratified by age, but not by KTC. The randomization scheme will be generated and reviewed by the study biostatistician and quality assurance staff and locked by them after approval. The investigation sites will assign eligible patients through an interactive internet-based computer system (IWRS) once a patient has satisfied the requirements to be included in the study. The IWRS randomization tool is part of the eCRF (Secutrial^®^, interActive Systems, Berlin, Germany). After screening for eligibility and assignment of the individual PID, each patient can only be assigned once to one of the treatment arms. Using the web-based tool will make it impossible for the study personnel to predict the assignment. The sites will record the time of randomization. Treatment in the assigned group starts in the first year after the kidney transplantation and ends in month 12 after randomization.

### Study visits

There are five planned study visits and two additional visits for capturing tacrolimus trough levels during the trial period.

### Baseline visit

At baseline (=visit 1), the patients will undergo routine medical examinations and central randomization will be performed to one of the two treatment groups in a 1:1 ratio. Different questionnaires will be assessed on paper or in electronic form. The established treatment will be continued until month 12, when the last control assessment will be performed. Investigators will be instructed to change immunosuppression only if necessary and based on clinical grounds. For any change in the immunosuppressive regimen, the investigator should explain this in the eCRF.

After the baseline visit, there will be four additional study visits during the 12-month treatment period.

Visit 2: 3 months ± 14 days after baseline

Visit 3: 6 months ± 21 days after baseline

Visit 4: 9 months ± 21 days after baseline

At the visits, 2–4 regular safety assessments (AEs/SAEs and AEs of special interest) and documentation of medication, BPAR, graft loss, and death will take place. The visit plan in section 4.7 shows the items to be evaluated at each time point. The different questionnaires will be assessed on paper or in electronic form. Most importantly, all visits and physician contact at the KTC, with the home nephrologists, the emergency unit, or admission to any hospital will be documented.

### Additional study visits

At two additional time points (at month 7.5 and month 10.5), tacrolimus trough levels will be captured and analyzed at the KTC. If blood is drawn outside the KTC, samples will be shipped to the laboratory to assess the patient’s tacrolimus trough level.

Additional visits in the transplant center may be necessary as per routine practice or for clinical reasons, such as a sign or symptom of drug toxicity/intolerance, an adverse event, or a suspected acute rejection episode, at any time during the study. During these additional visits, risk evaluation (blood, urine, and biopsy) with iBox Score can be performed when deemed necessary by the local investigator according to local policy or in situations such as allograft biopsy, anti-HLA DSA assessment, or deterioration of allograft function (estimated glomerular filtration rate, proteinuria). Furthermore, additional laboratory parameters may be obtained at study visits, if clinically indicated. In general, all patients should be followed according to local practice and as recommended by KDIGO clinical practice guidelines for the care of kidney transplant recipients (65).

### Dose of immunosuppressants and tacrolimus trough level monitoring

The doses of all immunosuppressants being administered to the patient will be recorded on the appropriate eCRF page at each study visit, as will any time a change in dosage is made and at any time a whole blood tacrolimus trough level is measured.

Whole blood, for tacrolimus trough level measurement, will be drawn within 30 minutes before the morning dose of tacrolimus at each study visit. Dose adjustments to maintain tacrolimus whole blood trough levels within the predefined therapeutic range will be based on local laboratory determinations. Each site should submit its local procedures for tacrolimus trough levels before beginning enrolment.

Investigators will be instructed to change immunosuppressant doses only if necessary (e.g., based on clinical grounds or trough levels far outside the target range).

### Study endpoints

The primary endpoint will be measured via seven indicators: BAASIS Score (adherent) after 12 months, IPV of tacrolimus trough levels < 30% between months 6 and 12, number of unplanned hospitalizations in 12 months, number of unplanned hospitalizations > 10 nights in 12 months, normal 24h blood pressure profile at 12 months, no *de novo* DSA at 12 months, and eGFR ≥ 45ml/min at 12 months. Each indicator of the primary endpoint can be defined as achieved (=1) or not achieved (=0) for each patient. The primary endpoint is calculated by an unweighted sum of the achievement of the indicators (min = 0, max = 7). Secondary endpoints were graft failure within 12 months, better iBox prognosis at 12 months, increase in quality of life (self-report) over 12 months, increase in illness management (self-report) over 12 months, and incremental cost-effectiveness. In addition, acceptance of the patients and the telemedicine teams of the new telemedical approach will be measured.

*Adherence* will be determined by the BAASIS questionnaire (38) at month 12. Non-adherence is defined as “yes” to any of the questions. For patients without valid responses to the questionnaire, the response to the BAASIS questionnaire at month 9 will be imputed. Patients who did not respond to the questionnaire at months 9 and 12 will be considered non-adherent.

*Tacrolimus IPV* will be determined from tacrolimus trough levels at months 6, 7.5, 9, 10.5, and 12. To determine tacrolimus trough levels at months 7.5 and 10.5, patients will undergo a blood draw at home (either nephrologist or a general practitioner) or at a KTC according to the patient’s decision. If necessary, whole blood will be shipped by mail to the transplant center for central evaluation. For patients with tacrolimus levels of less than 3 (except for patients with graft loss or switch to another immunosuppressant), high tacrolimus IPV will be assumed.

*Hospitalization* is defined as an unplanned hospital admission resulting in an overnight stay where the length of stay is at least 24 hours. All events leading to an emergency room visit with a stay under 24 hours will not be classified as hospitalization. For a hospitalization that is considered planned, the patient must not have had signs or symptoms of a worsened disease or need for intensified therapy. The date of admission must be arbitrary. Planned hospitalizations can be due to cardiovascular or non-cardiovascular reasons, e.g., those for diagnostic procedures, elective interventions (like a device battery change), or planned operations. For a hospitalization to be considered unplanned, the patient must present with new symptoms (except accidents) and/or worsening of existing symptoms with the need for immediate admission into a hospital for intensified diagnostic tests and therapy (66).

*A normal 24h RR profile* for adults is defined as ≤ 135/≤ 85 mmHg (18) on average with at least 12h duration of 24h RR measurement and for children with blood pressure below the 95^th^ percentile. For patients without a valid 24h RR profile, their ambulatory blood pressure at month 12 will be imputed.

Good *eGFR* will be defined as eGFR ≥ 45 ml/min as determined by CKD-EPI (67) or Schwartz formula (68) at month 12. For patients without available eGFR at 12 months, their last available eGFR value will be carried forward.

### Death

Patient survival will be calculated from the date of transplantation to the date of death for any cause.

### Graft loss

Graft survival will be calculated from the date of transplantation to the start date of chronic renal replacement therapy or listing for re-transplantation.

### Acute allograft rejection

The diagnosis of BPAR (Banff grade ≥ 1A) will be based on histological grading using the current Banff criteria for renal allograft pathology [79]. A new episode of acute rejection will not be considered to have occurred until at least 30 days have elapsed since the last treatment for rejection. If less than 30 days have elapsed, additional therapy will be considered as reinitiation of treatment for an ongoing episode. Suspected rejections that are not proven by biopsy, but are treated with a course of anti-rejection therapy, will be defined as “treated rejections”.

### Recommendation on the indication and timing of graft biopsies

The indication for kidney transplant biopsies is at the discretion of the investigator. Unless a biopsy is medically contraindicated, it is strongly recommended to perform a kidney biopsy under the following circumstances: any unexplained rise in creatinine ≥25% compared to baseline in absence of an obvious clinical cause for rising creatinine (e.g., ureteral obstruction, renal artery stenosis, dehydration, urosepsis, polyoma virus infection, or drug toxicity); in any case of clinical suspicion for rejection with/without an unexplained decrease in urinary output, fever, and graft tenderness; as soon as possible after starting treatment for suspected rejection; at development or worsening of proteinuria.

*Surveillance biopsies* are at the discretion of the investigator(s) and will be read locally. All data from protocol biopsies will be collected within the eCRF. Results of pre-transplant biopsies will be captured in the eCRF.

All biopsies should be read locally and classified according to the most recent Banff classification. In addition, biopsies may be read in a central laboratory at a later stage and documented within the eCRF. In the case of any BPAR, it is strongly recommended to perform local HLA antibody testing.

*Graft failure* is defined as the initiation of chronic dialysis for a period of more than 30 days, allograft nephrectomy, listing for re-transplantation, or death with a functioning graft following randomization. Graft survival will be calculated from the date of randomization to the date of graft failure.

### Development of *de novo* donor- specific anti-HLA antibodies

Anti-HLA antibodies and dnDSAs will be analyzed locally throughout the trial at pre-specified visits, at the time of any suspected acute rejection, or according to local protocols. Any newly developed donor-specific HLA antibody with an MFI >1,000 will be classified as DSA. The development of dnDSAs at month 12 post-randomization is part of the primary combined endpoint. In addition, the incidence of anti-HLA antibodies at baseline and the incidence and time to development of *de novo* anti-HLA antibodies and dnDSAs will be calculated.

### Hypertension

Hypertension in adults is defined by the latest treatment guideline from the European Society of Cardiology (69). The interpretation of blood pressure levels depends on the patient’s age; risk constellation due to additional diseases, such as diabetes, chronic kidney disease, or coronary artery disease; and how the antihypertensive therapy has been accepted. Blood pressure that is too high starts with “high normal”, whereas all levels from grade 1 should lead to the immediate initiation of therapy. For definition, see above. The cutoff values for adults apply only to adolescents aged 16 years and older. Blood pressure of pediatric patients will be evaluated as follows: hypertension is defined in children as blood pressure (BP) above the 95th percentile for age, sex, and height. Grade 1 hypertension corresponds to BP values >95th percentile up to the 99th percentile + 5 mmHg, while Grade 2 hypertension is defined as BP >99th percentile + 5 mmHg. Only children above 3.5 years will be included and the percentiles from the KIGGS report will be used as the reference.

### Post-transplant diabetes mellitus

For the analysis of PTDM at month 12, only patients with no medical history of diabetes before transplantation will be included. Diabetes will be assessed through measurement of fasting plasma glucose and HbA1c and the need for any antidiabetic agents (oral or insulin) (70). PTDM is defined by the need for any antidiabetic agent (oral or insulin) and/or HbA1c > 48 mmol/mol (> 6.5%) at month 12 in a patient with no prior medical history of diabetes before transplantation. In addition, post-transplant hyperglycemia and newly developed PTDM within 12 months after randomization will be assessed. Post-transplant hyperglycemia is defined by a fasting plasma glucose level ≥ 126 mg/dL and/or afternoon capillary glucose level ≥200mg/dl with or without insulin requirement or the need for an oral antidiabetic agent in a patient with no prior medical history of diabetes. Newly developed PTDM is defined as PTDM in patients without diabetic medication at randomization. Finally, the incidence of HbA1c elevation will be determined by an increase a) > 42 mmol/mol (> 6.0%), b) > 48 mmol/mol (> 6.5%), and c) > 53 mmol/mol (> 7.0%) at month 12 after randomization.

### Infections

All infections diagnosed by the investigator will be captured on specific infection CRF pages within the AE documentation chapter. The investigator will classify the severity (mild, moderate, severe, life-threatening, or fatal), type of infection (viral, bacterial, fungal, and other), and the seriousness of the infection (serious adverse event).

Opportunistic infections are defined as any CMV infection or viremia, BKV infection or viremia, or PJP.

### CMV

It is strongly recommended to provide prophylaxis and therapy measures for CMV according to the Third International Consensus Guidelines on the Management of Cytomegalovirus in Solid-Organ Transplantation (13 57)

CMV disease is defined as one or more of the following:

- Fever > 38°C for at least 2 days;
- New or increased malaise;
- Leukopenia;
- ≥ 5% atypical lymphocytes;
- Thrombocytopenia;
- Elevation of hepatic transaminases (ALT or AST) to 2 x the upper limit of normal;
- Evidence of CMV in blood by viral culture, antigenemia, or a DNA/RNA-based assay;
- No other cause of symptoms/signs identified.

Tissue-invasive CMV infections include symptoms/signs of organ dysfunction plus detection of CMV in affected tissue by culture, immunohistochemically analysis, or *in situ* hybridization. CMV-DNAemia is defined as asymptomatic (no CMV disease, no tissue invasive infection, or criteria of infection) CMV replication in blood

Assessment within the study:

- Nucleic acid testing (NAT) will be performed at randomization, and months 6, 9, and 12 after transplantation for safety purposes;
- Additional monitoring of CMV DNAemisis due to the discretion of the investigator(s);
- Monitoring by NAT testing is strongly recommended in any case of suspected CMV infection.

All data on CMV DNAemia and infection will be documented in the eCRF.

*BKPyV vDNAeimia and BKPyV virus infection, and BKPyV nephropathy.* BK vDNAemmia is defined as the presence of elevated BK virus loads detected by standard NAT in the local laboratory. BKPyV virus infection is defined as clinical symptoms (e.g., elevated creatinine or BKV nephropathy) in the presence of BK viremia. BKV nephropathy is histologically proven BKV infection of the graft [17, 97]. In addition, BKPyV viremia as such and the degree of elevated creatinine will be recorded.

### Cardiovascular events

A cardiovascular major adverse cardiac event (MACE), stroke, or symptomatic peripheral artery disease is named MACCE (major adverse cardiac and cerebrovascular event) and defined as cardiac death, non-fatal MI, or coronary revascularization procedure. The history of all cardiovascular events will be captured at randomization and all cardiovascular events will be recorded throughout the study after randomization as AEs/SAEs. The estimated 7-year risk of major adverse cardiovascular events and mortality will be calculated according to Soveri et al. [86] according to age (years), creatinine (mg/dl), total time on renal replacement therapy (months), number of transplants, presence of coronary heart disease (yes/no), smoking status (previous (yes/no), current (yes/no), diabetes mellitus (yes/no), and LDL-cholesterol (mmol/l).

### Predict4Health (iBOX) survival probabilities

The iBox survival probabilities (33) will be generated using eight parameters, including baseline characteristics (time from transplant to evaluation), functional parameters (eGFR and protein/creatinine ratio), immunological parameters such as the presence of circulating anti-HLA donor specific antibodies, and histological parameters [biopsy findings including microcirculation inflammation (glomerulitis -g Banff score and peritubular capillaritis -ptc Banff score), interstitial inflammation and tubulitis (i and t Banff scores), transplant glomerulopathy (cg Banff score), and interstitial fibrosis/tubular atrophy (IFTA Banff score)]. To account for missing variables and allow adaptation at individual centers, derived probabilities will be generated using the diagnosis of ABMR, TCMR, BK virus-associated nephropathy, or recurrence of end-stage renal disease (ESRD) instead of Banff scores. Thus, three types of risk evaluation will be generated based on available parameters, which will allow flexibility across centers:

- iBox#1: Functional (time from transplant to evaluation + eGFR + proteinuria) evaluation;
- iBox#2: Functional + immunological (DSA) evaluation;
- iBox#3: Functional + immunological + histological (biopsy) evaluation.

The iBox survival probabilities and evolution in time will give the clinician additional information to support their clinical decision to manage the patient. It is expected to give them earlier notice of any deviation or degradation in the allograft.

### Malignancy

Any malignant disease that occurs after transplantation will be recorded and the incidence will be calculated.

### Anemia

Anemia is defined as a hemoglobin value below normal and/or the use of erythropoietin-stimulating agents. The incidence of anemia will be determined throughout the study at and after randomization, and at months 6, 9, and 12 post-transplant. Within the final analysis, the incidence of low hemoglobin values <10g/dl or <11g/dl will be determined throughout the study at and after randomization, and at months 6, 9, and 12 post-transplant. Similarly, the incidence of the use of erythropoietin-stimulating agents will be determined throughout the study at and after randomization, and at months 6, 9, and 12 post-transplant.

### Leucocytopenia/Neutropenia

Leukopenia is defined as a leucocyte/neutrophil value below normal and/or the use of colony-stimulating factors. Within the final analysis, the incidence of low leucocyte values of < 4.0/nl, < 3.5/nl, < 3.0/nl, or < 2.5/nl will be determined throughout the study at and after randomization and at months 6, 9, and 12 post-transplant. The incidence of neutropenia < 1.5/nl will be determined throughout the study at and after randomization and at months 6, 9, and 12 post-transplant. The incidence of the use of colony stimulating factors will be determined throughout the study after randomization.

### Statistical analysis plan

For the data analysis, cross-sectional and longitudinal analytical methods will be employed using established statistical software. Longitudinal regression models (e.g., Mixed-Model with Repeated Measures (MMRM) will be used, allowing flexible modeling of endpoints while incorporating potential confounders should differences between the intervention group (IG) and control group (CG) exist in relevant influencing variables despite randomization. Through a multi-level approach, the clustering of individuals within the transplant centers (differences in intervention effect by center) can be accounted for, and the analyses can be additionally adjusted for confounders at the transplant center level (e.g., number of transplants per year, experience with telemedicine). Longitudinal analyses will be applied for the endpoints of improved quality of life and improved disease management. Depending on the scale level of the dependent variables, linear or logistic regressions will be calculated. For the incremental cost-effectiveness analysis, the ratio of the cost difference (estimated additional costs of the new care model compared to standard care) to the benefit difference between IG (with new care) and CG (without new care) will be calculated (ICER).

## Discussion

This study will provide novel fundamental data on whether additional interventional telemedical management will lead to a higher chance for long-term graft survival, increase adherence and QoL, and reduce complications and healthcare costs after kidney transplantation. As intended by the German Government, this study has the potential to optimize post-transplant care in Germany. If successful, our program will be extended to other transplanted organs and to all regions of Germany and the costs of positively evaluated elements will be covered by public health insurance companies. If our study is successfully completed, it could provide valuable information for the post-transplant management of KTR. Several pitfalls were taken into consideration. Since younger people are more digitally native, we had a concern that older KTRs would be disadvantaged, however, we approached all age groups and did not find that older patients had problems. Since this study represents a holistic approach, a primary endpoint based on seven indicators was chosen as the intervention does not primarily focus on one of them, but may result in unique effects on these indicators for each patient. By not focusing on a single variable, we account for these potentially differential effects of smartNTx. In combination, achieving as many of these indicators as possible represents a valid measurement of stable kidney function, adequate illness behavior, and a good health situation for the patients.

## Trial status

The trial has been registered at ClinicalTrials.gov: NCT05897047. The results of the trial will be published independent from outcome and results. Negative results will be published as well. Recruitment started in May 2023 and finished in April 2025. The last patient in was on 30 April 2025 and the last patient out is expected on 30 April 2026. At the timepoint of initial submission (15.02.2024) 327 patients had been randomized. At the timepoint of submission of revision, 391 patients were randomized so the required number of recruited patients was reached.
